# Evolutionary analyses of polymeric immunoglobulin receptor (pIgR) in the mammals reveals an outstanding mutation rate in the lagomorphs

**DOI:** 10.3389/fimmu.2022.1009387

**Published:** 2022-11-18

**Authors:** Fabiana Neves, Patrícia de Sousa-Pereira, José Melo-Ferreira, Pedro J. Esteves, Ana Pinheiro

**Affiliations:** ^1^ CIBIO-UP, Centro de Investigação em Biodiversidade e Recursos Genéticos, Universidade do Porto, InBIO, Laboratório Associado, Campus Agrário de Vairão, Vairão, Portugal; ^2^ BIOPOLIS Program in Genomics, Biodiversity and Land Planning, CIBIO, Vairão, Portugal; ^3^ School of Life Sciences, University of Dundee, Dundee, United Kingdom; ^4^ Departamento de Biologia, Faculdade de Ciências, Universidade do Porto, Porto, Portugal; ^5^ CITS - Centro de Investigação em Tecnologias de Saúde, CESPU, Gandra, Portugal

**Keywords:** evolution, european rabbit, positive selection, immune system, fc receptors

## Abstract

**Background:**

The transcytosis of polymeric immunoglobulins, IgA and IgM, across the epithelial barrier to the luminal side of mucosal tissues is mediated by the polymeric immunoglobulin receptor (pIgR). At the luminal side the extracellular ligand binding region of pIgR, the secretory component (SC), is cleaved and released bound to dimeric IgA (dIgA), protecting it from proteolytic degradation, or in free form, protecting the mucosa form pathogens attacks. The pIgR was first cloned for rabbit in early 1980’s and since then has been described for all vertebrates, from fish to mammals. The existence of more than one functional pIgR alternative-spliced variant in the European rabbit, the complete pIgR as other mammals and a shorter pIgR lacking two SC exons, raised the question whether other lagomorphs share the same characteristics and how has the *PIGR* gene evolved in these mammals.

**Results:**

To investigate these questions, we sequenced expressed pIgR genes for other leporid genus, *Lepus* spp., and obtained and aligned pIgR sequences from representative species of all mammalian orders. The obtained mammalian phylogeny, as well as the Bayesian inference of evolutionary rates and genetic distances, show that Lagomorpha pIgR is evolving at a higher substitution rate. Codon-based analyses of positive selection show that mammalian pIgR is evolving under strong positive selection, with strong incidence in the domains excised from the rabbit short pIgR isoform. We further confirmed that the hares also express the two rabbit pIgR isoforms.

**Conclusions:**

The Lagomorpha pIgR unique evolutionary pattern may reflect a group specific adaptation. The pIgR evolution may be linked to the unusual expansion of IgA genes observed in lagomorphs, or to neofunctionalization in this group. Further studies are necessary to clarify the driving forces behind the unique lagomorph pIgR evolution.

## Introduction

The polymeric immunoglobulin receptor (pIgR) is a transmembrane glycoprotein required for the transcytosis of polymeric IgA (pIgA) and to a lesser extent polymeric IgM (pIgM) across mucosal epithelial cells (1). pIgR is composed by an N-terminal ligand binding domain, a single membrane spanning region and a short cytoplasmic C-terminal tail. Polymeric immunoglobulins (pIg) produced by plasma cells located in the lamina propria underlying the epithelium can covalently bind to pIgR expressed in the basolateral surface of epithelial cells. pIgR alone or attached to the pIg and pIg-containing immune complexes is endocytosed and transcytosed through a series of intracellular vesicles to the apical surface. At the mucosal surface the ligand binding portion of the pIgR, known as the secretory component (SC), is cleaved off and released in a free form or as part of the complex with the pIg (1). The complex pIg-SC is known as secretory immunoglobulin, and the presence of secretory IgA (SIgA) in respiratory secretions is essential for mucosal immunity (2). The transcytosis of pIgA by pIgR ensures a continuous delivery of SIgA to the epithelial surface and mucosal secretions, together with intracellular neutralization and excretion of antigens and pathogens (3). Besides that, the SC has several N-glycans able to bind bacterial and host factors, reducing infection and inflammation (4, 5).

Association of SC with IgA has been shown to enhance the stability and effector function of pIgA, especially by providing protection from proteolytic degradation by bacterial proteases (6, 7). A SC-like polypeptide is also seen associated to the polymeric IgT in rainbow trout, the polymeric IgX in *Xenopus*, and the polymeric IgA in birds, suggesting that all vertebrate pIgRs participate on the transport of pIgs into external secretions (8–10).

The pIgR is the most ancient Fc receptor in vertebrates (8–10). While the mammals’ pIgR has five extracellular Ig-like domains, the most primitive form of pIgR found in teleost fish has only 2 extracellular domains, homologous to domains 1 and 5 of mammalian pIgR, and the pIgR from amphibians and birds lacks the homolog of domain 2, showing only 4 extracellular domains (11–13). Regarding mammals, only rabbits and bovines were shown to express an alternative-spliced variant lacking domains 2 and 3, which interestingly are expressed by a single exon (exon 4), unlike the other Ig-like extracellular domains which are encoded by individual exons (14–16). The first domain seems to be essential for binding polymeric IgA and IgM, containing similar complementarity-determining regions (CDRs) to the immunoglobulin variable domains (17). The interchange of these regions between human and rabbit showed a similar efficiency at binding to pIgA, however, the human CDR2 region is required to bind to pIgM (18). In some species, such as humans and cows, pIgR participates in the transport of both pIgA and IgM, while in other species, such as the rabbit and rodents, only pIgA is transported. The presence of the charged residue Glu at position 53 on the CDR2 is shared by several species besides humans, including orangutan, cow and pig, while species whose pIgR binds pIgA exclusively have a polar, uncharged residue at this position (Asn in mouse and rat) or a deletion (in rabbit) (1, 18).

The order Lagomorpha includes two families, Leporidae (rabbits and hares) and Ochotonidae (pikas), and is the sister group to Rodentia. These two orders, which have diverged at approximately 82 million years ago (mya) (19), constitute the superorder Glires, the sister group of the superorder Euarchonta that includes Primata, Scandentia and Dermoptera (20). The rabbit immune system has been widely studied revealing some uniquenesses such as the preferential usage of only one variable heavy chain (VH) gene in VDJ rearranjements (21) or having at least 15 immunoglobulin’s A (IgA) (22), characteristics that seem to extend to other Lagomorphs (23–26). The existence of more than one functional pIgR alternative-spliced variant in the European rabbit, raised the question whether other lagomorphs share this same characteristic and of how has the PIGR gene evolved in these mammals.

## Material and methods

### RNA extraction, *PIGR* amplification and sequencing

Total RNA was extracted from gut tissue samples stored in RNAlater at -20°C from European rabbit (subspecies *Oryctolagus cuniculus cuniculus* and *Oryctolagus cuniculus algirus*) and two species of hares (*Lepus europaeus *and *Lepus timidus*); one individual for each rabbit subspecies and hare species was used. These samples belong to the CIBIO/InBIO, Vairão, Portugal, tissue collection and have been previously used for the successful amplification of other expressed genes (27, 28). Total RNA was extracted using the RNeasy Mini Kit (Qiagen, Hilden, Germany) according to the manufacturer’s protocol, followed by first-strand cDNA synthesis with the SuperScriptTM III Reverse Transcriptase Kit (Invitrogen) using 1 μg RNA. The *PIGR* coding region was amplified by PCR using primers designed on the available European rabbit sequence (NM_001171045.1): Forward 5’ GCAGCCCAGGCCTAGTG 3’ and Reverse 5’ CTAGGCCTCCTTGGGGCCATC 3’, located to the 5’ UTR and 3’ UTR regions, respectively. PCR amplification was performed using Phusion with annealing temperature of 60°C for 15 sec extension, for 35 cycles. PCR products were purified (NucleoSpin Gel and PCR Clean-up kit, Macherel-Nagel, Germany) and cloned into the pGEM-T Easy vector system II (Promega. Madison. WI, USA). For each species, at least fifteen clones were selected. Sequencing was performed on an ABI PRISM 310 Genetic Analyser (PE Applied Biosystems).

### Phylogenetic analysis

Publicly available sequences for mammalian *PIGR* were obtained from GenBank (http://www.ncbi.nlm.nih.gov/genbank/). In total, 96 species representative of Artiodactyla, Cetacea, Perissodactyla, Carnivora, Chiroptera, Primata, Rodentia, Lagomorpha, Marsupialia and Monotremata, were included in the analyses (**Table 1**;- accession numbers are given in **Supplementary Material**: **Table 1**). The sequences obtained for the leporid *PIGR* coding region were aligned with the mammalian *PIGR* sequences using CLUSTAL W (29) as implemented in BioEdit v7.2.5 (30), and corrected manually as to respect the exon boundaries. The obtained alignment is given in **Supplementary Material**: Data 1.

**Table 1:** Number of species for each mammalian lineage used in this study.

Mammalian lineage	Number of species
Artiodactyla	6
Carnivora	16
Cetacea	6
Chiroptera	7
Marsupialia	5
Monotremata	2
Lagomorpha	6
Perissodactyla	4
Pholidota	1
Primata	24
Proboscidae	1
Rodentia	16
Sirenia	1
Tubulidentata	1

The full list of species and sequence accession numbers are given in **Supplementary Table 1**.

MEGA version X software (31) was used to construct a Maximum likelihood (ML) phylogenetic tree and to calculate genetic distances. The phylogenetic tree was constructed using the GTR+G+I model of nucleotide substitution, determined to be the best fitting model to our dataset by the Model Selection option in MEGA version X software (31). Node support was determined from 1000 bootstrap replicate trees. This software was also used to calculate the nucleotide distances using the maximum composite likelihood method, uniform rates among sites, heterogeneous rates among lineages and pairwise deletion of gaps options. N-linked glycosylation sites were estimated using the online tool NetNGlyc 1.0 Server, with + indicating a potential to reach the 0.5 threshold, and ++ to reach the 0.75 threshold (32). The nucleotide substitution rate variation among different pIgR domains from Leporidae and Ochotonidae was estimated in DnaSp version 6.12 (33). Sliding window analysis were performed with a window length of 250 nucleotides and a step size of 12 nucleotides along the nucleotide sequence alignment and plotting the differences as averages. Sites with alignment gaps were not counted.

### Nucleotide evolutionary rates

The evolutionary rates were further inferred using the Bayesian method implemented in BEAST v1.10.4 (34) under an uncorrelated relaxed clock model with a lognormal distribution (35). This relaxed clock allows variation of evolutionary rates across lineages. These analyses were calibrated using normally distributed priors for 14 dates of most recent common ancestors of monophyletic groups retrieved from TimeTree (36), with a standard deviation of 2: Mammalia (180 Mya), Theria (160 Mya), Placentalia (99 Mya), Boreotheria (94 Mya), Euarchontoglires (87 Mya), Glires (80 Mya), Scrotifera (79 Mya), Ferae (78 Mya), Afrotheria (78 Mya), Marsupialia (78 Mya), Euungulata (75 Mya), Certatiodactyla (64 Mya), Lagomorpha (51 Mya) and Monotremata (47 Mya). Posterior probabilities were determined using the Yule tree prior and a GTR+I+G nucleotide substitution model. Independent runs of 50,000,000 generations were performed, and convergence was assessed using Tracer v1.7 (37). Final estimates were based on the combined results of three replicate runs, discarding the first 10% as burn-in.

### Codon-based analyses of positive diversifying selection

To evaluate the incidence of positive selection on the mammalian pIgR evolution, we compared the rate per-site of nonsynonymous substitution (dN) to the rate per-site of synonymous substitutions (dS) in a maximum likelihood (ML) framework using three different methods. As each method employs unique algorithms, and hence has advantages and drawbacks, we only considered those codons identified by a minimum of two of the ML methods as being positively selected codons (PSC) (38–40). The obtained ML phylogenetic tree was constrained as to reflect the accepted mammalian phylogeny (41) and used as working topology in these analyses.

Using the CODEML program (PAMLX) (42, 43) we compared two alternative models—M8, which allows for codons to evolve under positive selection (dN/dS > 1) and M7, which does not (dN/dS ≤ 1). The analyses were performed with the F3x4 model of codon frequencies and were run twice to guarantee convergence. The models were compared using a likelihood ratio test with 2 degrees of freedom (44, 45). Codons under positive selection for model M8 were identified using a Bayes Empirical Bayes approach (46) and considering a posterior probability of more than 90%.

We then used four methods for detecting positively selected codons available on the DataMonkey web server (47): the Single Likelihood Ancestor Counting (SLAC) model, the Fixed Effect Likelihood (FEL) model, the Mixed Effects Model of Evolution (MEME) and the Fast Unbiased Bayesian Approximation (FUBAR). Each method was run three times to ensure consistent results. The best fitting nucleotide substitution model was first determined by the automatic model selection tool available on the server.

To verify that recombination was not providing a false assumption of positive selection (48–50), we used the GARD method from the DataMonkey web server (47) to screen the dataset. The results did not show evidence of recombination.

## Results

The existence of different rabbit pIgR transcripts has been known for a long time, and it was well established that they are the product of alternative splicing of a unique gene. In the shorter rabbit pIgR transcript the lack of domains 2 and 3, both encoded by the rabbit *PIGR* exon 4, does not affect binding to the pIgA, while the deletion of those domains abrogates the interaction between the pIgA and the human pIgR. This indicates a possible species adaptation, allowing the rabbit pIgR to be functional in the absence of these domains. However, few species were shown to express an alternative-spliced variant of the pIgR, raising the doubt if close related species to the European rabbit share this characteristic, and of how has the rabbit pIgR evolved.

### Mammalian pIgR evolution

**Figure 1:**
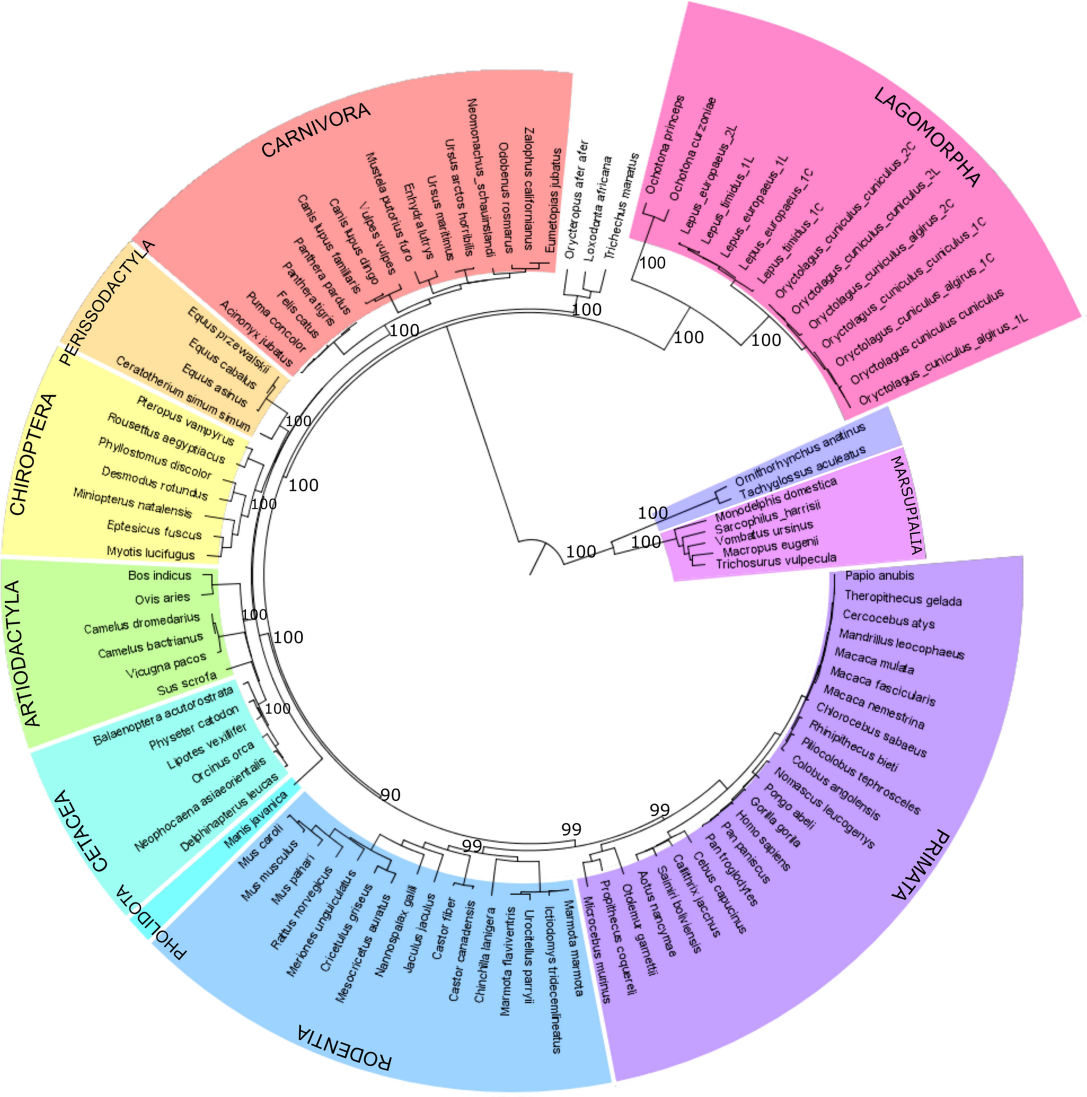
Phylogenetic tree of mammalian pIgR gene. Maximum likelihood (ML) method and the GTR+G+I model of nucleotide substitution were used to obtain the pIgR phylogenetic tree. Mammalian orders are highlighted in different colors. Bootstrap values are indicated near most relevant branches. For Lagomorpha sequences the last letter in the name identifies the long and short isoform that we sequenced, L identifies the long isoform and C the short isoform.

The *PIGR* coding region was amplified from extracted mRNA from different leporids, including European rabbit (subspecies *Oryctolagus cuniculus cuniculus* and *Oryctolagus cuniculus algirus*), and hare (*Lepus europaeus* and *Lepus timidus*) and further sequenced. The obtained leporid sequences were aligned with pIgR sequences from representatives of major mammalian orders, including two pika species (*Ochotona princeps* and *O. curzoniae*), and phylogenetically analysed. The obtained pIgR ML phylogeny generally conforms to the accepted mammalian phylogeny (41) with one notable exception (**Figure 1**). Instead of grouping with the Rodentia as in the mammalian phylogeny (41), the Lagomorpha group appears as a basal branch to the Eutherian mammals (**Figure 1**; 100% bootstrap). Additionally, the Lagomorpha two families, Leporidae and Ochotonidae, ancestral branches are longer compared to the other Eutherian mammals’ groups (**Figure 1**). These results suggest that the Lagomorpha pIgR has evolved at a higher substitution rate compared to other mammalian orders.

**Figure 2:**
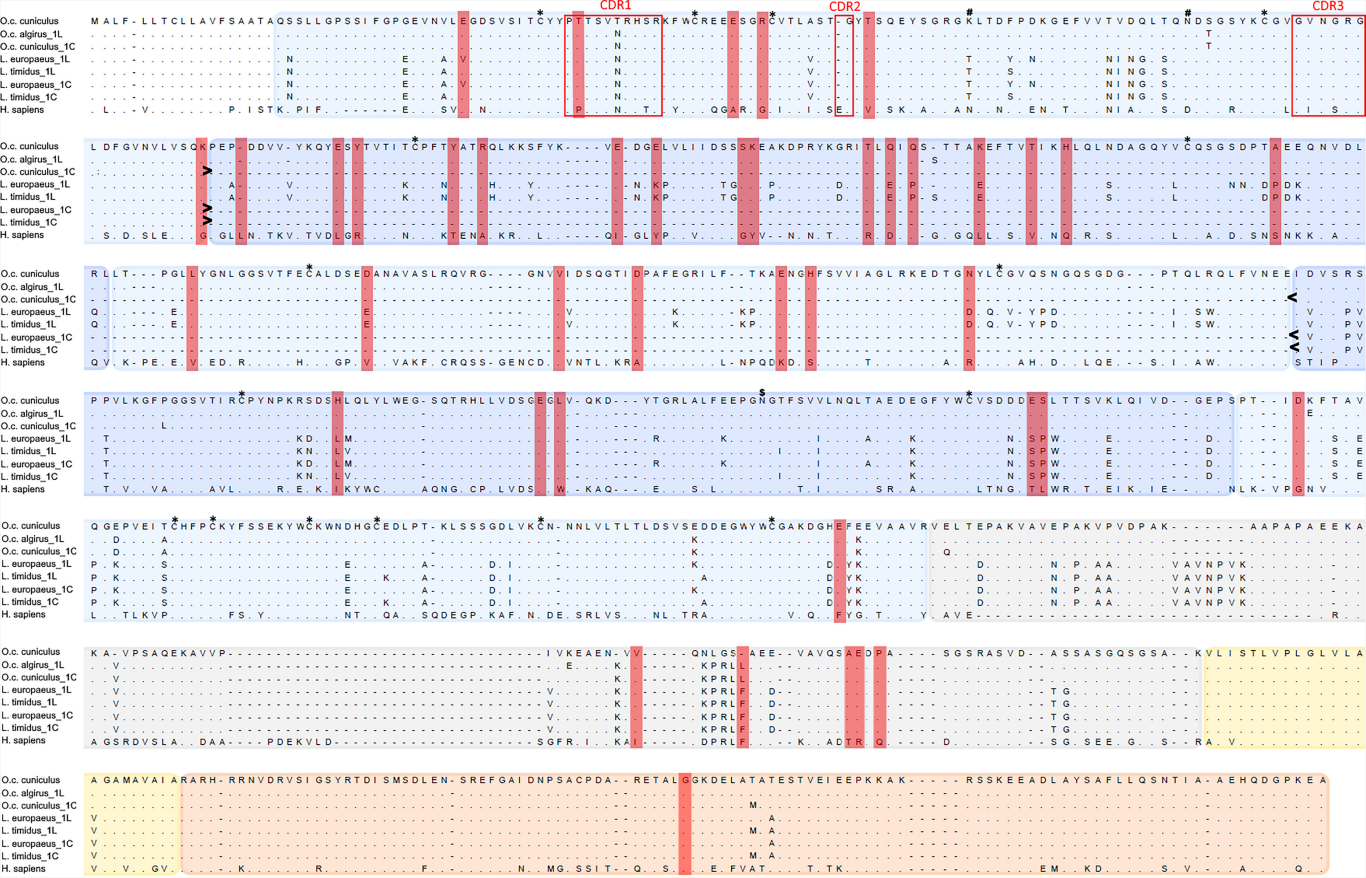
Alignment of leporid and human pIgR amino acid sequences. Shown are the pIgR amino acid sequences for the two European rabbit subspecies (*O.c. cuniculus* and *O.c. algirus*), the two studied hare species (*L. europaeus* and *L. timidus*) and Human (*H. sapiens*); for leporids, 1L identifies the long isoform and 1C the short isoform. The deletion of Ig like domains 2 and 3 in the leporids short isoforms is indicated between > and <. The PSC’s identified for the mammalian pIgR are highlighted in red rectangles. Protein domains are shaded in different colors: Ig like domains in two alternating blue accents, the linker spacer in grey, the transmembrane domain in yellow and the cytoplasmic tail in orange. The Ig like domain 1 CDRs are within red boxes. Conserved Cys residues are marked with * above the alignment. N-glycosilation sites are marked above the alignment: # indicates European rabbit glycosylation sites (51) and $ shows the rabbit and hare glycosylation site.

To evaluate the incidence of positive selection on mammalian pIgR evolution we compared two disparate models implemented in CODEML. The model that allows sites to evolve under positive selection (M8) showed a significantly better fit than the model that did not (M7) (lnL M7/lnL M8 =−59437.14/−59246.86; −2ΔlnL = 380; *α* < 0.001; **Supplementary Material Table 2**), revealing evidence for positive selection to be acting on mammalian pIgR. Comparing the sites recognized by each of the five employed methods led to the identification of 43 PSCs. Of these 43 PSCs, 37 are located in the Ig like domains, with a greater incidence on Ig like domain 2 (16 PSC’s), 5 in the linker spacer and one in the cytoplasmic tail (**Figure 2**). Curiously, the two Ig like domains found to be under greater incidence of positive selection are excised from rabbit and cow pIgR short isoforms (14, 16).

### Lagomorph pIgR evolution

**Figure 3:**
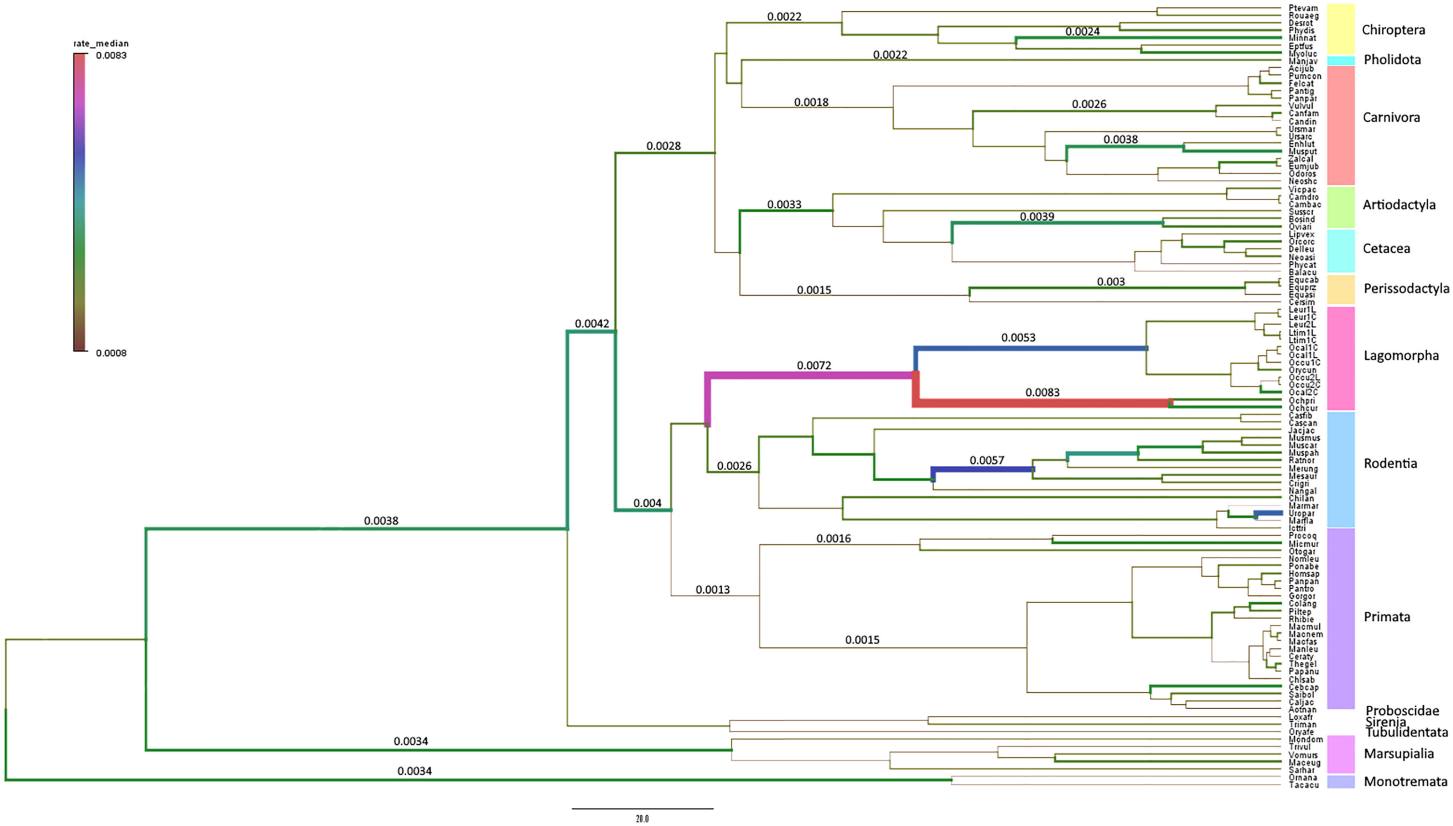
Branch-specific evolutionary rates (substitutions/site/million years) for *PIGR* estimated using BEAST v1.10.4 (34). The median of the high posterior density distribution is mapped onto the mammalian phylogenetic tree. The thickness and color of branches vary according to the inferred rate. The mammalian orders are indicated in different colors in a vertical bar to the right of the tree.

To confirm our hypothesis, that the Lagomorpha pIgR has evolved at a higher substitution rate compared to other mammalian orders, we conducted a Bayesian inference of evolutionary rates for all studied mammalian lineages. This analysis showed that the Lagomorpha, Ochotonidae and Leporidae ancestral branches have substantially higher substitution rates than the other Eutherian mammals’ groups or even Monotremata (Lagomorpha 0.0072 substitutions/site/million years, Ochotonidae 0.0083 substitutions/site/million years, Leporidae 0.0053 substitutions/site/million years, substitutions/site/million years; 0.0034 substitutions/site/million years Monotremata) (**Figure 3**).

The calculated nucleotide genetic distances for the pIgR sequences also suggest that the Lagomorpha pIgR is evolving at a higher rate than other mammals. The Lagomorpha pIgR genetic distances to other Eutherian mammals’ orders range between 0.2409 and 0.3862 (**Table 2**) while genetic distances between Eutherian mammals’ orders range between 0.0655 and 0.2898 (**Table 2**). The Lagomorpha is the sister group to Rodentia, having diverged at approximately 80 mya (36). Together, these groups constitute the superorder Glires, which diverged from Primata at approximately 89 mya (36). Nonetheless, the genetic distances obtained between Lagomorpha pIgR and Rodentia or Primata pIgR (0.2409 and 0.2693, respectively) were two to three-fold higher than the genetic distance obtained between Rodentia and Primata pIgR (0.1009; **Table 2**).

**Table 2:** Nucleotide genetic distances between the pIgR of mammalian lineages.

	Lagomorpha	Primata	Chiroptera	Rodentia	Perissodactyla	Cetacea	Proboscidae	Sirenia	Artiodactyla	Pholidota	Carnivora	Tubulidentata	Monotremata
**Lagomorpha**													
**Primata**	0.2693												
**Chiroptera**	0.2779	0.1374											
**Rodentia**	0.2409	0.1009	0.1318										
**Perissodactyla**	0.3142	0.1727	0.1144	0.1683									
**Cetacea**	0.3203	0.1628	0.1163	0.1595	0.1561								
**Proboscidae**	0.3650	0.2137	0.1978	0.2133	0.2337	0.2340							
**Sirenia**	0.3736	0.2318	0.2266	0.2284	0.2532	0.2620	0.1529						
**Artiodactyla**	0.2825	0.1355	0.0984	0.1361	0.1318	0.0655	0.2131	0.2378					
**Pholidota**	0.3623	0.2056	0.1688	0.2099	0.2152	0.1920	0.2753	0.3072	0.1718				
**Carnivora**	0.2922	0.1374	0.0824	0.1294	0.1249	0.1141	0.2129	0.2324	0.0983	0.1720			
**Tubulidentata**	0.3862	0.2126	0.2073	0.2088	0.2368	0.2339	0.1908	0.1976	0.2133	0.2898	0.2012		
**Marsupialia**	0.4802	0.3875	0.3792	0.3675	0.4250	0.4080	0.4279	0.4554	0.3812	0.4737	0.3899	0.4229	
**Monotremata**	0.5892	0.4956	0.4859	0.4875	0.5189	0.5064	0.5206	0.5390	0.4680	0.5464	0.5016	0.5465	0.4638

These were calculated in MEGA X software using the maximum composite likelihood method, uniform rates among sites, heterogeneous rates among lineages and pairwise deletion of gaps options. The leporids short pIgR transcripts were not used in this analysis.

The calculated genetic distances among Lagomorpha species are also indicative of a high mutation rate for the Ochotonidae and Leporidae pIgR. The genetic distances between rabbit or hares and pikas pIgR (0.3881 to 0.4045; **Table 3**), species that diverged at approximately 50 mya (36), are five-fold higher than the genetic distances between rabbit and hares pIgR (0.0722 to 0.0779; **Table 3**), members of the Leporidae family which diverged at approximately 12 mya (36). Concurrently, the nucleotide identity between European rabbit or hares pIgR and Ochotona pIgR is of 58% and amino acid homology is of 32%. As for the leporid pIgR it is conserved, with nucleotide identity of 92% and amino acid homology of 86% between European rabbit and the hare species pIgR.

**Table 3:** Nucleotide genetic distances between pIgR alleles of studied Lagomorpha species.

	*O. princeps*	*O. curzoniae*	*L.europaeus*_2L	*L.timidus*_1L	*L.europaeus*_1L	*O.c.cuniculus*_2L	*O.c.cuniculus*
***O. curzoniae***	0.0906						
***L.europaeus*_2L**	0.3988	0.4045					
***L.timidus*_1L**	0.3979	0.4044	0.0086				
***L.europaeus*_1L**	0.3988	0.4044	0.0103	0.0121			
***O.c.cuniculus_*2L**	0.3908	0.3928	0.0703	0.0698	0.0707		
***O.c.cuniculus***	0.3936	0.3940	0.0765	0.0751	0.0779	0.0232	
***O.c.algirus*_1L**	0.3881	0.3884	0.0722	0.0739	0.0736	0.0179	0.0109

These were calculated in MEGA X software using the maximum composite likelihood method, uniform rates among sites, heterogeneous rates among lineages and pairwise deletion of gaps options.

The high divergence between Leporidae and Ochotonidae pIgR raised the question of whether the rabbit and Ochotona pIgR proteins could be the product of alternatively spliced variants. To clarify this question, we aligned the full genomic sequences of European rabbit, hare and pika and found that the encoded pIgR sequence is flanked by conserved introns across lagomorphs (**Supplementary Material: Data 2**). Thus, the divergence observed between Leporidae and Ochotonidae pIgR is truly the product of high substitution rates in the coding sequence.

**Figure 4:**
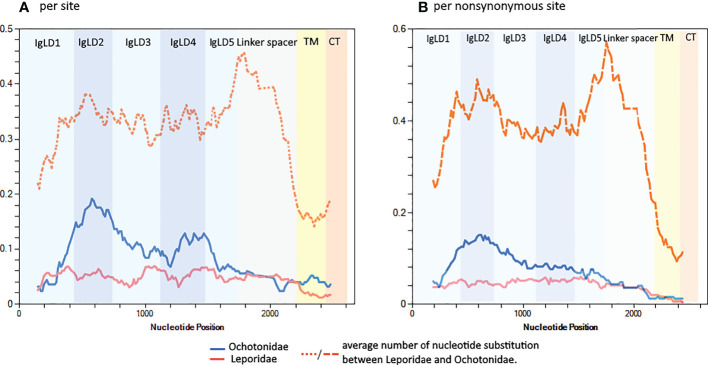
Sliding window along the pIgR nucleotide sequences showing the nucleotide diversity for Leporidae and Ochotonidae for **(A)** all sites and for **(B)** nonsynonymous sites. All Lagomorph sequences used in this study, except the Leporidae short transcripts, were used in this analysis. The analysis was performed in DnaSP version 6.12 with a window length of 250 nucleotides and a step size of 12 nucleotides. The pIgR domains are indicated.

Next, we asked which pIgR regions are experiencing higher substitution rates. The analysis of the nucleotide diversity along the pIgR domains shows that, overall, this parameter is higher for Ochotonidae than for Leporidae (**Figure 4**). The average number of nucleotide substitution per site between Leporidae and Ochotonidae, is, for the Ig like domains and linker spacer approximately the double than that for the conserved transmembrane and cytoplasmic tail domains with peaks in the linker spacer and Ig like domain 2 (**Figure 4**), showing that these domains are the regions where the divergence between Leporidae and Ochotonidae pIgR is occurring.

Focusing only on non-synonymous sites shows a similar pattern, a higher nucleotide diversity for Ochotonidae, with a peak for Ig like domain 2. The non-synonymous sites nucleotide divergence between Leporidae and Ochotonidae for the Ig like domains and linker spacer is, on average, approximately four times higher than that observed for the transmembrane and cytoplasmic tail and shows peaks in the Ig like domain 2 and linker spacer domain (**Figure 4**). This analysis shows that a high proportion of the nucleotide substitutions between Ochotonidae and Leporidae are non-synonymous and confirms that the Ochotonidae and Leporidae pIgR amino acid sequence is highly divergent for the Ig like and linker spacer domains while being identical in the transmembrane and cytoplasmic tail. (see **Supplementary Material: Data 3** for an alignment of Leporidae, Ochotonidae and Human pIgR amino acid sequence).

### Leporid pIgR characterization

Our PCR’s of leporids pIgR yielded two products of approximately 2400bp and 1700bp for each analysed individual. Sequencing confirmed that the two products correspond to the two described European rabbit transcripts, the long, full pIgR gene, and the short transcript, lacking Ig like domains 2 and 3, for both subspecies of European rabbit and the two hare species (**Figures 1**, **2**), evidencing that these short transcripts have evolved at least in a leporid ancestor.

All the cysteine residues involved in structurally important disulfide bonds are conserved in leporid species, and the CDR regions of domain 1 are well conserved (**Figure 2**). The prediction for N-glycosylation sites showed that, while the European rabbit has three sites for N-linked glycosylation sites the hares have one or no sites for N-glycosylation (419 NGTF + for *L. europaeus* and none for *L. timidus*) (**Figure 2**).

## Discussion

The European rabbit immune system has some uniqueness among which are the existence of 15 IgA’s (22) and the expression of two functional pIgR alternative-spliced variants, one full length and a shorter isoform lacking Ig like domains 2 and 3 (14). In this study, we asked how has the mammalian pIgR evolved and additionally we verified if other leporids would share the two European rabbit isoforms.

Our analyses of positive selection evidenced that mammalian pIgR is evolving under strong positive selection as many immune system genes are (38–40, 52). The domain with more sites under positive selection is Ig like domain 2 (16 out of the 43 PSC’s). This is not entirely surprising as domains 1, 4 and 5 have been described as highly conserved in mammals (1). This result does highlight that Ig like domain 2 should be particularly important in the function or conformation of the pIgR in mammals and raises the question of how has the lagomorph pIgR evolved to express a shorter isoform lacking this domain. The combined deletion of domains 2 and 3 of human pIgR eliminated binding and transcytosis of pIgA across transfected MDCK cells. This suggests that Ig like domains 2 and 3 may be necessary for maintaining the correct orientation of Ig like domain 5 and allow disulfide bonding with the Cα3 domain of IgA (53–55). On the other hand, Ig like domain 1 of rabbit pIgR was found to bind pIgA with equivalent affinity to the full-length SC, and the deletion of Ig like domains 2 and 3 did not diminish binding to pIgA (56, 57). These species-specific differences in pIgA-pIgR interactions may provide a functional explanation for species-specific differences in alternative splicing of the exon encoding Ig like domains 2 and 3 in pIgR mRNA, since both the full-length rabbit pIgR and the alternative-spliced variant lacking the Ig like domains 2 and 3 can bind and lead to transcytosis of IgA dimers *in vitro* (58, 59).

The phylogenetic tree of the mammalian pIgR further suggested that the Lagomorph pIgR has been evolving at a higher substitution rate than other mammals. To confirm this later hypothesis, we ran a Bayesian inference of evolutionary rates for all studied mammalian lineages and calculated the nucleotide genetic distances. The obtained results concur to show that the ancestral Lagomorpha pIgR has a higher substitution rate than other eutherian mammals, and that this acceleration in the substitution rate persists in the Ochotonidae and Leporidae branches. Among vertebrate Fc receptors, the pIgR is the most evolutionary ancient. A primitive pIgR emerged in bony fishes (60–62), and probably evolved by duplication of the Ig-like domains that constitute the extracellular region (60). While the mammals pIgR has five extracellular Ig-like domains, the most primitive form of pIgR found in teleost fish has only 2 extracellular domains, homologous to domains 1 and 5 of mammalian pIgR, and the pIgR from amphibians and birds lacks the homolog of domain 2, showing only 4 extracellular domains (11–13). Phylogenetic studies support the coevolution of *PIGR* and the mucosal *IGH* genes, for instance, the distance between the trout IgT and the frog IgX is similar to the distance between the trout and the frog pIgR, suggesting an adaptation of the pIgR to the transition from IgT to IgX as the dominant mucosal Ig isotype (60). Considering that Lagomorpha uniquely have multiple IgA copies (23) and that the unique acceleration of the substitution rate of the pIgR occurred before the Lagomorpha radiation, it is tempting to associate both events. Thus, the IgA expansion would have driven the acceleration in the pIgR substitution rate or vice-versa. However, known pIgR functions make it difficult to establish this association, so we cannot exclude the possibility that the pIgR has acquired a new function that could be related to the IgA expansion. Further studies are necessary to understand what has driven the acceleration in the substitution rate of Lagomorpha pIgR. Interestingly, other immune system genes, like TLR5 gene is also evolving at higher rate in lagomorphs than other mammalian orders (63). The TLR5 recognizes flagellin, the major protein of bacterial flagella, and triggers the immunologic responses for the clearance of the pathogen (64) among which is a TLR5 dependent IgA response (65, 66).

The high divergence between the Leporidae and Ochotonidae pIgR was also somewhat surprising. Since the two lagomorph families uniquely have multiple IgA copies (23), it would be interesting to compare the divergence between Leporidae and Ochotonidae pIgR and Leporidae and Ochotonidae IgA’s but, unfortunately, there are no available IgA sequences for pikas. Should the two families’ IgA divergence parallel the divergence we found for pIgR, then it would be a support for the hypothesis that the two genes are coevolving in this mammalian group. However, other immune system genes also seem to present significate evolutionary differences between leporids and ochotonids, the CCL16 is pseudogenized in Leporidae but not in Ochotonidae (67) and the IL17 seems to present a higher than expected divergence between Ochotonidae and Leporidae (68), and so this may be a regular pattern.

By sequencing the *PIGR* in leporids, we confirmed that, like the European rabbit, hares express the two pIgR isoforms, suggesting that this feature should have appeared at least in a Leporidae ancestral. Ig like domains 2 and 3 were shown to be essential for the human pIgR to bind pIgA. Their absence in the low molecular weight rabbit pIgR was, however, proven harmless regarding pIgA binding, since rabbit pIgR domain 1 alone efficiently binds dIgA (53, 56, 58). A smaller transcript, equally lacking Ig like domains 2 and 3, was identified in bovine, however, this shorter form is far less abundant than the full length bovine pIgR (16). On the contrary, the two rabbit transcripts are nearly equally expressed in different tissues, suggesting that the alternative splicing is not tissue specific (14), which might also apply for other leporids, remaining only unclear what controls the expression of the different transcripts. The leporids *PIGR* shows a high degree of nucleotide similarity and an equally high percentage of amino acid identity, presenting a high conservation of residues important for the structure and function of pIgR. The number of possible N-linked glycosylation sites for the European rabbit was previously determined, and only three sites were identified, whereas seven sites were identified in the human pIgR (51, 69). Some of the sites for N-glycosylation identified for the rabbit pIgR vary depending on the allotype analyzed, however the degree or site of glycosylation does not significantly affect the binding of Ig like domain 1 to dIgA (69). In light of this observation, the very low or absence of glycosylation level seen for the hare pIgR may not affect its function.

pIgR proteins with fewer extracellular domains are found in other distant species, including teleost fish, amphibians and birds, however, those are not generated by alternative splicing, but simply have a shorter extracellular portion with domains that might represent the ancestral mammalian domains (11–13, 70). It is intriguing that the evolution of the mammalian pIgR lead to the formation of five extracellular Ig-like domains, followed by further adaptation in the leporid lineage that allowed the successful expression of a smaller but fully functional transcript. Therefore, further studies are necessary to determine the biological significance of the two forms of plgR found in these species.

## Data availability statement

The datasets presented in this study can be found in online repositories. The names of the repository/repositories and accession number(s) can be found in the article/**Supplementary Material**.

## Author contributions

FN carried out the laboratory work and analysed the data. PS-P analysed the data and drafted the manuscript. JM-F performed the evolutionary rates analyses. AP analysed the data and edited the manuscript. AP and PE conceived the study. All authors contributed to the article and approved the submitted version.

## Funding

This work was co-funded by the project NORTE-01-0246-FEDER-000063, supported by Norte Portugal Regional Operational Programme (NORTE2020), under the PORTUGAL 2020 Partnership Agreement, through the European Regional Development Fund (ERDF). The authors also acknowledge research suport via the project PTDC/BIA-OUT/29667/2017 – POCI-01-0145-FEDER-029667, co-funded via national funds through FCT—Foundation for Science and Technology— and EU funds European Regional Development Fund (ERDF). FCT also supported the post-doctoral fellowships of AP (SFRH/BPD/117451/2016) and the Investigator grants of JMF (2021.00150.CEECIND) and PJE (CEECIND/CP1601/CT0005).

## Conflict of interest

The authors declare that the research was conducted in the absence of any commercial or financial relationships that could be construed as a potential conflict of interest.

## Publisher’s note

All claims expressed in this article are solely those of the authors and do not necessarily represent those of their affiliated organizations, or those of the publisher, the editors and the reviewers. Any product that may be evaluated in this article, or claim that may be made by its manufacturer, is not guaranteed or endorsed by the publisher.
