# Supplementary material for: Evolutionary analyses of polymeric immunoglobulin receptor (pIgR) in the mammals reveals an outstanding mutation rate in the lagomorphs
Source: Front Immunol. 2022 Nov 18;13:1009387. doi: 10.3389/fimmu.2022.1009387 (PMC9716071; doi:10.3389/fimmu.2022.1009387)
Supplement: Supplementary file 1 [file DataSheet_1.zip › Supplementary_Material.docx]

Supplementary Material

# Supplementary Data

**Supplementary Data 1.** Alignment of the mammalian pIgR sequences used in this study. For lagomorpha sequences the last letter in the name identifies the long and short isoform that we sequenced, L identifies the long isoform and C the short isoform.

**Supplementary Data 2.** European rabbit (*Oryctolagus cuniculus cuniculus*), hare (*Lepus europaeus*) and pika (*Ochotona princeps*) *PIGR* full genomic nucleotide sequence alignment.

**Supplementary Data 3.** European rabbit (*Oryctolagus cuniculus cuniculus*), hare (*Lepus europaeus*), pikas (*Ochotona princeps*) and human pIgR amino acid sequence alignment.

# Supplementary Tables

**Supplementary Table 1.** Genbank accession number of the sequences used in this study

| **Species** | **Common name** | **Order-Family** | **Accession number** |  |
| --- | --- | --- | --- | --- |
| *Sarcophilus harrisii* | Tasmanian devil | Dasyuromorphia - Dasyuriudae | XM_012547830.2 | predicted |
| *Vombatus ursinus* | Wombat | Diprotodontia - Vombatidae | XM_027871238.1 | predicted |
| *Trichosurus vulpecula* | Common brushtail possum | Diprotodontia - Phalangeridae | AF091137.1 | mRNA |
| *Macropus eugenii* | Tammar wallaby | Diprotodontia -  Macropodidae | AF317205.1 | mRNA |
| *Monodelphis domestica* | Gray short-tailed opossum | Didelphimorphia - Didelphidae | XM_007481294 | predicted |
| *Tachyglossus aculeatus* | Short-beaked echidna | Monotremata - Tachyglossidae | XM_038749170 | predicted |
| *Ornithorhynchus anatinus* | Platypus | Monotremata - Ornithorhynchidae | XM_007670271 | predicted |
| *Loxodonta africana* | Elephant | Proboscidea – Elephantidae | XM_010590270.2 | predicted |
| *Orycteropus afer afer* | Aardvark | Tubulidentata - Orycteropodidae | XM_042782515.1 | predicted |
| *Trichechus manatus* | West Indian manatee | Sirenia - Trichechidae | XM_004375200.1 | predicted |
| *Nannospalax galili* | Palestine mole-rat | Rodentia -Spalacidae | XM_017795646.1 | predicted |
| *Jaculus jaculus* | lesser jerboa | Rodentia - Dipodidae | XM_004663454.1 | predicted |
| *Castor fiber* | Eurasian beaver | Rodentia - Castoridae | KU948186.1 |  |
| *Castor canadensis* | American beaver | Rodentia - Castoridae | XM_020153100.1 | predicted |
| *Cricetulus griseus* | Chinese hamster | Rodentia – Cricetidae | XM_027417714.1 | predicted |
| *Mesocricetus auratus* | Golden hamster | Rodentia – Cricetidae | XM_005079862.3 | predicted |
| *Ictiodomys tridecemlineatus* | Thirteen-lined ground squirrel | Rodentia- Sciuridae | XM_013360378.2 | predicted |
| *Mus musculus* | House mouse | Rodentia – Muridae | NM_011082.3 | mRNA |
| *Mus pahari* | Gairdner's shrewmouse | Rodentia – Muridae | XM_021199094.1 | predicted |
| *Mus caroli* | Ryukyu mouse | Rodentia – Muridae | XM_021163773.1 | predicted |
| *Urocitellus parryii* | Arctic ground squirrel | Rodentia- Sciuridae | XM_026387498.1 | predicted |
| *Rattus norvegicus* | Brown rat | Rodentia – Muridae | NM_012723.4 | mRNA |
| *Meriones unguiculatus* | Mongolian gerbil | Rodentia – Muridae | XM_021640132.1 | predicted |
| *Marmota flaviventris* | Yellow-bellied marmot | Rodentia- Sciuridae | XM_027934636.1 | predicted |
| *Marmota marmota* | Alpine marmot | Rodentia- Sciuridae | XM_015483735.1 | predicted |
| *Chinchilla lanigera* | Long-tailed chinchilla | Rodentia – Chinchillidae | XM_005375408.2 | predicted |
| *Sus scrofa* | Pig | Artiodactyla – Suidae | XM_021102216.1 | predicted |
| *Bos indicus* | Indian cattle | Artiodactyla – Bovidae | XM_019976844.1 | predicted |
| *Camelus dromedarius* | Camel | Artiodactyla – Camelidae | XM_010991335.1 | predicted |
| *Camelus bactrianus* | Bactrian camel | Artiodactyla – Camelidae | XM_010963251.1 | predicted |
| *Vicugna pacos* | Llama | Artiodactyla – Camelidae | XM_006215460.2 | predicted |
| *Ovis aries* | Domestic sheep | Artiodactyla - Bovidae | XM_004013573.3 | predicted |
| *Equus cabalus* | Horse | Perissodactyla - Equidae | NM_001284539.1 | predicted |
| *Equus przewalskii* | Przewalskii’s horse | Perissodactyla - Equidae | XM_008541727.1 | predicted |
| *Equus asinus* | Donkey | Perissodactyla - Equidae | XM_014828879.1 | predicted |
| *Ceratotherium simum simum* | Southern white rhinoceros | Perissodactyla - Rhinoceratidae | XM_004425089.2 | predicted |
| *Balaenoptera acutorostrata* | Minke whale | Cetacea - Balaenopteridae | XM_007171562.2 | predicted |
| *Physeter catodon* | Sperm whale | Cetacea - Physeteridae | XM_007105464.2 | predicted |
| *Lipotes vexillifer* | Baiji | Cetacea - Lipotidae | XM_007470715.1 | predicted |
| *Orcinus orca* | Orca | Cetacea - Delphinidae | XM_004282419.1 | predicted |
| *Neophocaena asiaeorientalis* | Yangtze finless porpoise | Cetacea - Phocoenidae | XM_024750889.1 | predicted |
| *Delphinapterus leucas* | Beluga whale | Cetacea - Monodontidae | XM_022593570.1 | predicted |
| *Manis javanica* | Sunda pangolin | Pholidota - Manidae | XM_017669902.1 | predicted |
| *Phyllostomus discolor* | Pale spear-nosed bat | Chiroptera – Phyllostomidae | XM_028530977.1 | predicted |
| *Eptesicus fuscus* | Big brown bat | Chiroptera – Vespertilionidae | XM_008154172.2 | predicted |
| *Myotis lucifugus* | Little brown bat | Chiroptera – Vespertilionidae | XM_014460947.2 | predicted |
| *Pteropus vampyrus* | Large flying fox | Chiroptera – Pteropodidae | XM_011384989.2 | predicted |
| *Rousettus aegyptiacus* | Egyptian fruit bat | Chiroptera – Pteropodidae | XM_016129288.1 | predicted |
| *Desmodus rotundus* | Vampire bat | Chiroptera – Phyllostomidae | XM_024575443.1 | predicted |
| *Miniopterus natalensis* | Natal Long-fingered Bat | Chiroptera – Miniopteridae | XM_016215978.1 | predicted |
| *Ursus arctos horribilis* | Grizzly bear | Carnivora – Ursidae | XM_026480734.1 | predicted |
| *Ursus maritimus* | Polar Bear | Carnivora – Ursidae | XM_008685076.1 | predicted |
| *Canis lupus dingo* | Dingo | Carnivora – Canidae | XM_025429745.1 | predicted |
| *Canis lupus familiaris* | Dog | Carnivora – Canidae | NM_001287152.1 | predicted |
| *Vulpes vulpes* | Red fox | Carnivora – Canidae | XM_025987313.1 | predicted |
| *Mustela putorius furo* | Ferret | Carnivora – Mustelidae | XM_004756188.2 | predicted |
| *Enhydra lutrys* | Sea otter | Carnivora – Mustelidae | XM_022508993.1 | predicted |
| *Panthera pardus* | Leopard | Carnivora – Felidae | XM_019430322.1 | predicted |
| *Panthera tigris* | Tiger | Carnivora – Felidae | XM_007096282.2 | predicted |
| *Felis catus* | Cat | Carnivora – Felidae | XM_006942944.3 | predicted |
| *Puma concolor* | Cougar | Carnivora – Felidae | XM_025921193.1 | predicted |
| *Acinonyx jubatus* | Cheetah | Carnivora – Felidae | XM_015079984.2 | predicted |
| *Neomonachus schauinslandi* | Hawaiian monk seal | Carnivora - Phocidae | XM_021682511.1 | predicted |
| *Odobenus rosmarus* | Walrus | Carnivora - Odobenidae | XM_004415535.1 | predicted |
| *Zalophus californianus* | California sea lion | Carnivora - Otariidae | XM_027613275.1 | predicted |
| *Eumetopias jubatus* | Steller sea lion | Carnivora - Otariidae | XM_028120050.1 | predicted |
| *Otolemur garnettii* | Small-eared Galago | Primata – Galagidae | XM_003792206.3 | predicted |
| *Macaca fascicularis* | Crab-eating macaque | Primata- Cercopithecidae | XM_005540664.2 | predicted |
| *Macaca nemestrina* | Southern pig-tailed macaque | Primata- Cercopithecidae | XM_011746914.2 | predicted |
| *Macaca mulatta* | Rhesus macaque | Primata- Cercopithecidae | XM_015117898.1 | predicted |
| *Cercocebus atys* | Sooty mangabey | Primata- Cercopithecidae | XM_012040214.1 | predicted |
| *Mandrillus leocophaeus* | Drill | Primata- Cercopithecidae | XM_011967046.1 | predicted |
| *Papio anubis* | Olive baboon | Primata- Cercopithecidae | XM_003893189.3 | predicted |
| *Theropithecus gelada* | Gelada | Primata- Cercopithecidae | XM_025400948.1 | predicted |
| *Chlorocebus sabaeus* | Green monkey | Primata- Cercopithecidae | XM_007988655.1 | predicted |
| *Rhinipithecus bieti* | Black snub-nosed monkey | Primata- Cercopithecidae | XM_017880880.1 | predicted |
| *Piliocolobus tephrosceles* | Ugandan red colobus | Primata- Cercopithecidae | XM_023186967.1 | predicted |
| *Colobus angolensis* | Angola colobus | Primata- Cercopithecidae | XM_011937581.1 | predicted |
| *Nomascus leucogenys* | Northern White-cheeked gibbon | Primata- Hylobatidae | XM_003272974.2 | predicted |
| *Pongo abeli* | Orangutan | Primata- Hominidae | NM_001131626.1 | mRNA |
| *Gorilla gorila* | Gorilla | Primata- Hominidae | XM_004028295.2 | predicted |
| *Homo sapiens* | Human | Primata- Hominidae | NM_002644.4 | mRNA |
| *Pan paniscus* | Bonobo | Primata- Hominidae | XM_003822909.2 | predicted |
| *Pan troglodytes* | Chimpanzee | Primata- Hominidae | XM_514153.6 | predicted |
| *Cebus capucinus* | Colombian White-faced capuchin | Primata- Cebidae | XM_017525490.1 | predicted |
| *Callithrix jacchus* | Common marmoset | Primata- Callitrichidae | XM_002760737.4 | predicted |
| *Saimiri boliviensis* | Black-capped squirrel monkey | Primata- Cebidae | XM_003930430.2 | predicted |
| *Aotus nancymae* | Nancy Ma’s night monkey | Primata- Aotidae | XM_021677317.1 | predicted |
| *Propithecus coquereli* | Coquerel’s sifaka | Primata - Indriidae | XM_012638850.1 | predicted |
| *Microcebus murinus* | Gray mouse lemur | Primata - Cheirogaleidae | XM_012760568.1 | predicted |
| *Oryctolagus cuniculus cuniculus* | European rabbit | Lagomorpha - Leporidae | NM_001171045.1 | mRNA |
| *Ochotona prínceps* | American pika | Lagomorpha - Ochotonidae | XM_004578930.2 | predicted |
| *Ochotona curzoniae* | Plateau pika | Lagomorpha - Ochotonidae | XM_040985572.1 | predicted |
| **Sequences obtained in this study** | | | |  |
| *Oryctolagus cuniculus cuniculus_1C* | European rabbit | Lagomorpha - Leporidae | OP121165 | mRNA |
| *Oryctolagus cuniculus cuniculus_2C* | European rabbit | Lagomorpha - Leporidae | OP121162 | mRNA |
| *Oryctolagus cuniculus cuniculus_2L* | European rabbit | Lagomorpha - Leporidae | OP121163 | mRNA |
| *Oryctolagus cuniculus algirus_1C* | European rabbit | Lagomorpha - Leporidae | OP121166 | mRNA |
| *Oryctolagus cuniculus algirus_1L* | European rabbit | Lagomorpha - Leporidae | OP121167 | mRNA |
| *Oryctolagus cuniculus algirus_2C* | European rabbit | Lagomorpha - Leporidae | OP121164 | mRNA |
| *Lepus europaeus_1L* | Brown hare | Lagomorpha - Leporidae | OP121159 | mRNA |
| *Lepus europaeus_1C* | Brown hare | Lagomorpha - Leporidae | OP121160 | mRNA |
| *Lepus europaeus_2L* | Brown hare | Lagomorpha - Leporidae | OP121157 | mRNA |
| *Lepus timidus_1C* | Mountain hare | Lagomorpha - Leporidae | OP121161 | mRNA |
| *Lepus timidus_1L* | Mountain hare | Lagomorpha - Leporidae | OP121158 | mRNA |

**Supplementary Table 2.** Phylogenetic tests of positive selection for the mammalian pIgR.

^1^ PSC (Positively Selected Codons) – only the codons identified by at least two of the ML methods were considered to be positively selected codons

| **Test of selection** | | | **Sites under selection identified by different methods** | | | | |  |  |
| --- | --- | --- | --- | --- | --- | --- | --- | --- | --- |
| **lnL M7/**  **lnL M8** | **-2lnΔLc** | **significance** | **PAML M8** | **MEME** | **FEL** | **SLAC** | **FUBAR** | **PSC^1^** | |
| -59437.14/  -59246.86 | 380 | ** (p<0.001) | 141,163,180,  184,187,192,  193,194,202,  205,206,208,  210,216,217,  224,243,325,  371,374,411,  422,528,578,  638,656,677,  699,700,704,  705,707,713 | 35,39,51,67,70,81,103,104,  127,137,141,144,151,154,  163,166,169,180,183,184,  186,187,193,194,199,206,  208,210,216,221,224,230,  234,243,254,259,261,263,  279,280,281,282,307,319,  321,322,324,325,326,329,  340,361,375,376,385,398,  400,401,402,411,415,419,  420,421,422,423,424,425,  430,471,472,477,495,500,  511,542,545,555,556,562,  563,578,636,639,644,653,  659,660,678,680,681,682,  692,693,701,702,703,704,  705,706,707,708,732,733,  736,756,759,774,800,810,  812,825,826,827,829,830,  832,844,848,832,844,848 | 15,39,51,137,141,  151,163,166,180,  184,208,216,221, 224,243,261,263,  279,299,340,401,  471,472,495,578,  679,682,693,705,  812 | 39,51,70,81,  83,141,151,  163,166,180,  184,206,208,  216,224,243,  261,279,340,  359,401,682,  705 | 110,141,151,163,166,180,184,206,208,216,224,243,261,279,319,340,359,401,682,705 | 39,51,67,70,  81,137,141,  151,153,163,  166,180,184,193,194,  206,208,210,  216,221,224,  243,261,  279,299,307,322,325,  340,401,  411,422,424,  471,472,495,  578,682,693,  704,705,707,  812 | |
